# Cost-effectiveness analysis of benmelstobart and anlotinib plus chemotherapy versus standard chemotherapy in first-line treatment for extensive-stage small cell lung cancer: Perspectives from the United States and China

**DOI:** 10.1371/journal.pone.0331338

**Published:** 2025-09-26

**Authors:** Wenwang Lang, Jiangbo Wang, Ming Ouyang, Haiqing Zhao, Tingting Zhou, Lingyue Huang

**Affiliations:** Department of Pharmacy, Nanxishan Hospital of Guangxi Zhuang Autonomous Region, Guilin, China; Inha University Hospital, KOREA, REPUBLIC OF

## Abstract

**Background:**

Benmelstobart combined with anlotinib and chemotherapy has demonstrated significant clinical advantages in extending progression-free survival and overall survival compared to chemotherapy alone in patients with extensive-stage small-cell lung cancer (ES-SCLC). This is the first study to assess its cost-effectiveness from both the US payer and Chinese healthcare system perspectives.

**Method:**

A Markov state-transition model was utilized for the economic evaluation, reflecting both the perspectives of the US payer and the Chinese healthcare system. Baseline patient demographics and vital clinical data were obtained from the ETER701 trial. Costs and utilities were obtained from open-access databases and published literature. The primary outcomes evaluated were quality-adjusted life years (QALYs), incremental cost-effectiveness ratio (ICER), incremental net health benefit (INHB), and incremental net monetary benefit (INMB). The uncertainties of the model were addressed through probabilistic sensitivity analysis, one-way sensitivity analysis, and scenario analysis.

**Results:**

In the base-case scenario, adding benmelstobart and anlotinib to chemotherapy increased QALYs by 0.34 at an additional cost of $24,684.07, yielding an ICER of $71,559.84 per QALY. This exceeds the willingness-to-pay (WTP) threshold of $38,042.49 per QALY in China, making the treatment marginally cost-effective, with an INHB of −0.30 QALYs and an INMB of -$11,561.58. In the US, the treatment resulted in a QALY increase of 0.36, but incurred an additional cost of $151,052.04, leading to an ICER of $416,398.56 per QALY, surpassing the US WTP threshold of $150,000. 00.

**Conclusion:**

The combination of benmelstobart and anlotinib with chemotherapy is not a cost-effective first-line treatment option for ES-SCLC in either China or the US.

## Introduction

Lung cancer continues to be the leading cause of cancer-related deaths worldwide and is the second most frequently diagnosed cancer [1–3] Small cell lung cancer (SCLC) represents approximately 15% of all lung cancers and is characterized by rapid progression and early spread to other parts of the body. Most SCLC cases (80–85%) are diagnosed at an advanced stage, known as extensive-stage SCLC (ES-SCLC), with a poor prognosis [4]. Although ES-SCLC initially responds well to chemotherapy, almost all patients experience relapse within six months, resulting in a grim 5-year survival rate of less than 5% [5] The introduction of immune checkpoint inhibitors (ICIs) and platinum-based chemotherapy has shown promise in extending median survival rates.

Five phase III clinical trials have shown that adding ICIs such as atezolizumab, durvalumab, serplulimab, adebrelimab, or tislelizumab to chemotherapy significantly improves overall survival (OS) [6–12]. Moreover, anti-angiogenic therapies are known to enhance the effects of ICIs in various cancer types [13]. Normalizing blood vessels may improve immune cell infiltration and alter the tumor’s microenvironment [14]. Anti-angiogenic drugs, such as bevacizumab, sorafenib, apatinib, and cabozantinib, are widely used in tumor treatment. Anlotinib, also a multi-targeted anti-angiogenic drug [15], has shown potential synergy with ICI therapy and has been approved as a third-line treatment for ES-SCLC in China [16].

Benmelstobart, a humanized anti-programmed death-ligand 1 (PD-L1) antibody, has shown preclinical antitumor effects like other ICIs.

In the ETER701 phase 3 trial [17], a combination of benmelstobart and anlotinib with etoposide and carboplatin (EC) led to significant survival benefits compared to chemotherapy with EC alone as first-line treatment, which is a relatively optimal plan that should be selected first. The group receiving benmelstobart and anlotinib achieved a median OS of 19.3 months, a 7.4-month improvement over EC alone, marking the longest observed survival period. This group’s median progression-free survival (PFS) was 6.9 months, a 2.7-month extension compared to EC alone. Although the incidence of grade 3 or higher treatment-emergent adverse events (TEAEs) was slightly higher in the benmelstobart and anlotinib group (94.3%) compared to the EC group (89.0%), the difference was minimal.

Although benmelstobart and anlotinib have extended survival in patients with ES-SCLC when combined with chemotherapy, further research is needed to assess its cost-effectiveness and identify patient populations that would benefit the most. The high cost of benmelstobart raises questions regarding its financial accessibility to patients, highlighting the importance of developing a suitable pricing strategy. As a result, this study assessed the cost-effectiveness of benmelstobart and anlotinib plus EC as first-line treatment options for ES-SCLC in China and the US. It may be possible to optimize resource utilization by incorporating such evidence into clinical practices and reimbursement policies.

## Methods

### Model structure

**Fig 1:**
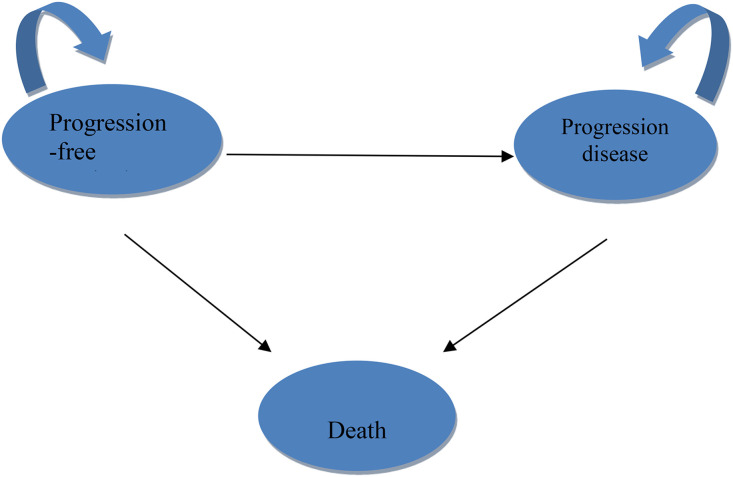
Markov model structure.

The consolidated health economic evaluation report standards statement (CHEERS) checklist was used to guide the design and execution of this study (S1 Table) [18]. The model outcomes were developed and evaluated using TreeAge Pro 2022 software (Williamstown, MA, USA) and R software (version 4.2.3, Vienna, Austria). A three-state Markov model, which included the following health states: PFS, progressive disease (PD), and death (**Fig 1**), was used. Patients entered the model in PFS state and could move to another state on the basis of transition probabilities as well as the transition direction.The simulation was conducted over 10 years, capturing more than 99% of mortality events in both treatment groups. The analysis was performed from two perspectives: the US payer’s perspective, which included only direct medical costs [19], and the Chinese healthcare systems, which included all healthcare-related expenses.

### Patients and intervention

The targeted participants were adults aged ≥ 18 years with histologically or cytologically confirmed ES-SCLC. The baseline characteristics of the patients were consistent with those observed in the ETER701 trial.

Eligible participants received etoposide (100 mg/m² intravenously on days 1–3 of each cycle) and carboplatin (administered at an area under the plasma or serum concentration-time curve of 5) on the first day of each three-week cycle for up to four cycles. Patients randomized to the experimental group received benmelstobart (1,200 mg intravenously) and anlotinib (12 mg orally once daily) on days 1–14 of each cycle. The control group was treated with a placebo. Efficacy evaluations were conducted every two cycles, following the protocol established in the ETER701 trial.

Among the participants, 105 (42.68%) in the benmelstobart and anlotinib plus chemotherapy groups and 176 (71.26%) in the placebo group received second-line treatments that were changed after the first-line regimen was proven to have failed. Assumptions regarding body surface area and creatinine clearance were based on data from previous studies [20].

The cost analysis for adverse events (AEs) was conducted using data from the ETER701 trial, focusing on grade 3 or 4 serious adverse events (SAEs) that occurred at a rate higher than 3%. For cost estimation purposes, it was assumed that all AEs occurred during the first treatment cycle. Tables 1 and 2 provide a detailed breakdown of the incidence rates of each AE [17].

**Table 1:** Key clinical input data(China).

Parameters		Baseline value	Range		Distribution	Reference
			Minimum	Maximum		
*Survival model for OS*						
Benmelstobart and anlotinib plus chemotherapy		Meanlog = 2.9149 Sdlog = 0.9062			Lognormal	[21]
Placebo plus chemotherapy		Shape = 2.4470 Scale = 12.8850			Loglogistic	[21]
*Survival model for PFS*						
Benmelstobart and anlotinib plus chemotherapy		Mu = 1.9133 Sigma = 0.6989 Q = −0.7736			Gengamma	[21]
Placebo plus chemotherapy		Shape = 4.4280 Scale = 5.0570			Loglogistic	[21]
*Drug cost, $/per cycle*						
Cost of Benmelstobart		3485.32	2788.26	4182.38	Gamma	Local charge
Cost of Tislelizumab		355.78	284.62	426.94	Gamma	Local charge
Cost of Anlotinib		563.24	450.59	675.89	Gamma	Local charge
Cost of Carboplatin		51.26	41.01	61.51	Gamma	Local charge
Cost of Cisplatin		35.03	28.02	42.04	Gamma	Local charge
Cost of Etoposide		6.63	5.30	7.96	Gamma	Local charge
Cost of Topotecan		246.69	197.35	296.03	Gamma	Local charge
Cost of the laboratory test		92.99	74.39	111.59	Gamma	[22]
PET-CT		912.28	729.82	1094.74	Gamma	[22]
Cost of end-of-life		1460.30	1168.24	1752.36	Gamma	[23,24]
Best supportive care		345.60	276.48	414.72	Gamma	[23]
Cost of drug administration per unit	Preventive medication per intravenous administration	93.93	75.14	112.72	Gamma	[23,24]
	Infusion fee per intravenous administration	1.86	1.49	2.23	Gamma	[23,24]
	Preventive medication	39.14	31.31	46.97	Gamma	[23,24]
*Proportion of receiving subsequent treatment*						
Benmelstobart and anlotinib plus chemotherapy group		42.68%	34.14%	51.22%	Beta	[21]
Placebo plus chemotherapy		71.26%	57.01%	85.51%	Beta	[21]
*Cost of AEs, $*						
Anaemia		138.75	111.00	166.50	Gamma	[22]
Decreased platelet count		1505.92	1204.74	1807.10	Gamma	[22]
Decreased neutrophil count		115.01	92.01	138.01	Gamma	[22]
Hypertension		120.00	96.00	144.00	Gamma	[25]
*Utilities*						
Utility of PFS		0.69	0.55	0.83	Beta	[26]
Utility of PD		0.60	0.48	0.72	Beta	[26]
Disutility estimates						
Anemia		0.073	0.058	0.088	Beta	[22]
Decreased platelet count		0.05	0.04	0.06	Beta	[22]
Decreased neutrophil count		0.20	0.16	0.24	Beta	[22]
Hypertension		0.12	0.10	0.14	Beta	[25]
*Risk for main AEs in Benmelstobart and anlotinib plus chemotherapy group*						
Anemia		23.98%	44.76%	67.14%	Beta	[21]
Thrombocytopenia		49.59%	2.82%	4.22%	Beta	[21]
Neutropenia		69.51%	13.04%	19.56%	Beta	[21]
Hypertension		15.45%	12.36%	18.54%	Beta	[21]
*Risk for main AEs in Placebo plus Chemotherapy group*						
Anemia		23.58%	44.76%	67.14%	Beta	[21]
Thrombocytopenia		35.77%	2.82%	4.22%	Beta	[21]
Neutropenia		68.70%	13.04%	19.56%	Beta	[21]
Hypertension		1.62%	1.30%	1.94%	Beta	[21]
Discount rate(China)		5%	4.00%	6.00%	Beta	
BMI/m2		1.72				
Weight/kg		65				
$1 = ¥7.0467		38,042.49				

OS: overall survival, PFS: progression-free survival, PD: progression disease, AE: adverse event, BMI: body mass index.

**Table 2:** Key clinical input data (US).

Parameters	Baseline value	Range		Distribution	Reference
		Minimum	Maximum		
*Drug cost, $/per cycle*					
Cost of Benmelstobart	8469.41	6775.53	10163.29	Gamma	Local charge
Cost of Anlotinib	1915.02	1532.02	2298.02	Gamma	Local charge
Cost of Tislelizumab	8640.00	6912.00	10368.00	Gamma	Local charge
Cost of Carboplatin	55.83	44.66	67.00	Gamma	[27]
Cost of Cisplatin	45.79	36.63	54.95	Gamma	[27]
Cost of Etoposide	62.76	50.21	75.31	Gamma	[27]
Cost of Topotecan	2720.26	2176.21	3264.31	Gamma	[27]
Cost of the laboratory test	111.65	89.32	133.98	Gamma	[27]
PET-CT	1769.89	1415.91	2123.87	Gamma	[27]
Cost of end-of-life	21603.00	17282.40	25923.60	Gamma	[28]
Best supportive care	1447.79	1158.23	1737.35	Gamma	[28]
Cost of drug administration first hour	142.55	114.04	171.06	Gamma	[29]
Administration intravenous, additional hour	30.68	24.54	36.82	Gamma	[29]
*Cost of AEs, $*					
Anaemia	7941.00	6352.80	9529.20	Gamma	[28]
Decreased platelet count	13105.00	10484.00	15726.00	Gamma	[28]
Decreased neutrophil count	13105.00	10484.00	15726.00	Gamma	[28]
Hypertension	15811.00	12648.80	18973.20	Gamma	[30]
Discount rate	3%	2.40%	3.60%	Beta	
BMI/m2	1.79				
Weight/kg	65				

AE: adverse event, BMI: body mass index

### Base-case analysis

The outcomes were overall survival in life years, quality-adjusted life-years (QALYs), incremental cost-effectiveness ratio (ICER), incremental net health benefits (INHB), and incremental net monetary benefits (INMB). Costs and utilities were discounted at an annual rate of 3% in the US and 5% in China [31,32]. For the Chinese analysis, all costs were inflation-adjusted to 2023 values using the local consumer price index and converted to US dollars based on an exchange rate of $1 = ¥7.0467.

The willingness-to-pay (WTP) threshold was defined as three times the 2023 per capita gross domestic product (GDP), in accordance with World Health Organization (WHO) guidelines [25,27]. This resulted in WTP thresholds of $38,042.49 for China and $150,000.00 for the US. Additionally, the analysis included calculations for the INHB and the INMB. The formulas used were: INHB (λ) = (μE1 - μE0) – (μC1 - μC0)/ λ = ΔE – ΔC/ λ and INMB (λ) = (μE1 - μE0) × λ – (μC1 - μC0) = ΔE × λ – ΔC. In these equations, μCi and μEi represent the costs and utility values associated with benmelstobart and anlotinib plus chemotherapy (i = 1) or placebo plus chemotherapy (i = 0), respectively, while λ denotes the WTP threshold.

### Clinical data input

**Fig 2:**
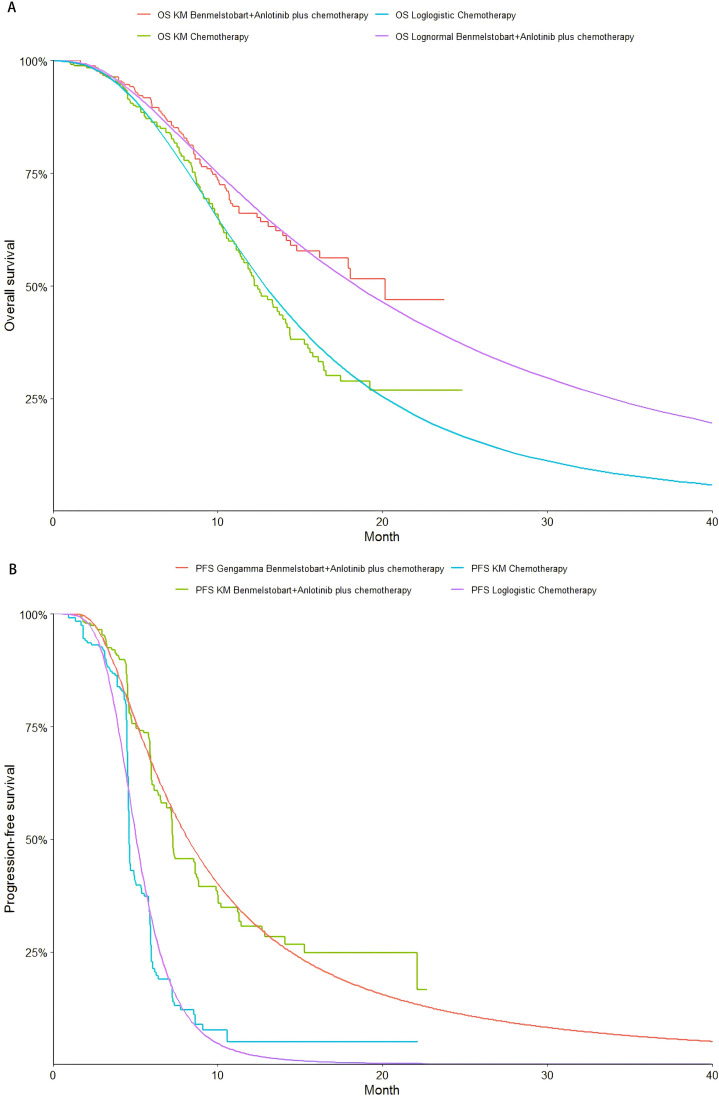
The Kaplan-Meier. (A) overall survival curves, (B) progression-free survival curves.

PFS and OS data points were extracted from survival curves using GetData (version 2.26; http://www.getdata-graph-digitizer.com/index.php). The extracted data were fitted to parametric survival models, including Exponential, Weibull, Weibull Proportional Hazards (Weibull PH), Gamma, Log-normal, Gompertz, Generalized Gamma, and Log-logistic distributions. Model selection was based on the Akaike Information Criterion (AIC) and Bayesian Information Criterion (BIC), supplemented by visual inspection. **Fig 2** and **Table 3** provide details on the survival model selection process. Shape parameters (g) and scale parameters (λ) were estimated for each survival model based on the fitting process and applied to reconstructed Kaplan-Meier curves using R (version 4.4.2, http://www.r-project.org) (**Table 1**, S2 and S3 Tables).

**Table 3:** The Akaike information criteria (AIC) and Bayesian information criteria (BIC).

Type of distribution	Benmelstobart and anlotinib plus chemotherapy (OS)		Placebo plus chemotherapy (OS)		Benmelstobart and anlotinib plus chemotherapy (PFS)		Placebo plus chemotherapy (PFS)	
	AIC	BIC	AIC	BIC	AIC	BIC	AIC	BIC
Exponential	1382.625	1386.050	1503.834	1507.272	1181.758	1185.183	1150.136	1153.574
Gamma	1373.174	1380.024	1449.605	1456.482	1181.420	1188.270	1026.2668	1033.143
Generalized gamma	1372.931	1383.206	1446.635	1456.949	1103.257	1113.532	987.626	997.940
Gompertz	1383.591	1390.441	1484.056	1490.933	1152.051	1158.901	1147.922	1154.798
Weibull	1375.636	1382.486	1458.289	1465.165	1183.599	1190.449	1080.105	1086.981
WeibullPH’	1375.636	1382.486	1458.289	1465.165	1183.599	1190.449	1080.105	1086.981
Log-logistic	1365.336	1372.185	1442.167	1449.044	1104.266	1111.116	943.475	950.352
Lognormal	1377.243	1384.093	1446.152	1453.028	1117.294	1124.144	993.907	993.907

OS: overall survival; PFS: progression-free survival; AIC: Akaike information criterion; BIC: Bayesian information criterion.

### Cost input

The analysis considered only direct medical costs, including drug costs, laboratory test fees, PET-CT scans, prophylactic medications for intravenous administration, best supportive care, end-of-life expenses, drug administration costs, subsequent treatment expenses, and costs associated with managing grade 3 and 4 adverse events (AEs). Drug pricing data were obtained from public databases and local pricing schedules, whereas other cost information was collected from previously published studies and relevant literature. Since benmelstobart and anlotinib patient assistance programs are currently available, the potential price reductions for both benmelstobart and anlotinib were factored into the analysis. Owing to the absence of available US prices for benmelstobart and anlotinib, the prices of these drugs were estimated through a comparative approach [33]. Drugs approved by both the FDA and NMPA were examined, and a price index was applied to estimate their US costs. The pricing of SCLC drugs tislelizumab and toripalimab in the United States is 80% of that of durvalumab [34]. The price of the lung cancer drug bevacizumab in the US is 3.4 times that of China. Using the same ratio, the cost of benmelstobart was estimated at 80% of the PD-L1 inhibitor atezolizumab, while anlotinib was priced at 3.4 times its cost in China. Drug doses were aligned with the ETER701 study protocol, and costs per treatment cycle were calculated based on local pricing information (**Tables 1 and 2**) [22–24,28,29,30,35,36].

### Utility inputs

Utility values were used to approximate the QoL of the patients, reflecting the impact of disease-related health on a scale from 0 (worst health) to 1 (optimal health). The mean health utility values for the PFS and PD states in this analysis were 0.673 and 0.473, respectively, based on published data (**Table 1**) [26]. Disutility values for AEs of grade ≥ 3 were also considered in these analyses [22,30].

### Scenario analysis

Owing to the significant uncertainties regarding the assumptions and parameter sources used in this study, scenario analyses were conducted. From this process, a price index was calculated, leading to an estimated US price for benmelstobart (1200 mg) of $8,469.41 and $3,485.32 in China. These analyses explored the potential cost-effectiveness by varying the drug price from $0 to $8,000 per 1,200 mg against the WTP thresholds of $38,042.49 in China and $150,000.00 in the US.

### Sensitivity analysis

The uncertainty of the model result was predicted using a range of sensitivity analyses. One-way sensitivity analyses (OWSA) were conducted within 20% of baseline values using different parameter values within defined ranges through the use of established approaches to assess the effects of individual parameters on ICER values. To conduct probabilistic sensitivity analyses (PSA), 10,000 Monte Carlo simulations were performed, enabling the simultaneous assessment of changes in several parameters. Cost-effectiveness acceptability curves for individual treatment strategies were evaluated as the most cost-effective at the WTP threshold.

## Results

### Base-case analysis

Over ten years, the base-case analysis revealed that the combination of benmelstobart and anlotinib with chemotherapy yielded an additional 0.99 QALYs at an incremental cost of $41,247.31. In contrast, the chemotherapy-only group saw an additional health beneft of 0.64 QALYs, costing $16,563.24. A comparative analysis with the combination therapy group demonstrated a mean incremental effect of 0.34 QALYs at an extra cost of $24,684.07. ICER for combination therapy compared to chemotherapy alone was calculated at $71,559.84 per QALY (**Table 4**). When assessed against China’s WTP threshold of $38,042.49 per QALY, combination therapy was found to be less cost-effective than chemotherapy alone, with an INHB of −0.30 QALYs and an INMB of $-11,561.58 (**Table 4**). In the US, the ICER for combination therapy reached $41,6398.56 per QALY, surpassing the US WTP threshold of $150,000.00 per QALY (**Table 4**), with an INHB of −0.64 QALYs and an INMB of $-96,638.29 compared to chemotherapy alone at the same WTP threshold (**Table 4**).

**Table 4:** The base case analysis.

Treatment	Cost	QALY	Incremental cost	Incremental QALY	INHB	INMB	ICER
Benmelstobart and anlotinib plus chemotherapy(China)	41247.31	0.99	24684.07	0.34	−0.30	−11561.58	71559.84
Chemotherapy(China)	16563.24	0.64					
Benmelstobart and anlotinib plus chemotherapy(US)	278741.24	1.02	151052.04	0.36	−0.64	−96638.29	416398.56
Chemotherapy(US)	127689.20	0.66					

QALY: Quality-adjusted life year, ICER: Incremental cost-effectiveness ratio, INMB: the incremental net monetary benefits, INHB: the incremental net health benefits.

### Price simulation

**Fig 3:**
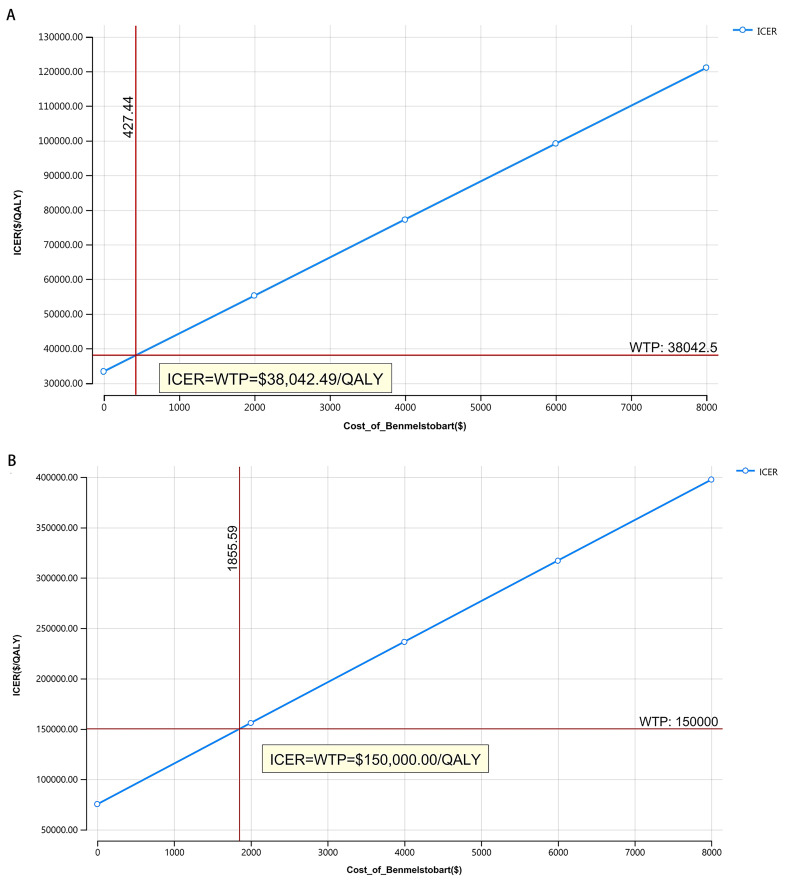
Price simulation. (A) China, (B) The US.

The outcomes of the price simulation, as shown in **Fig 3**, illustrate that, as the price of benmelstobart ranged from $0 to $8,000.00 per 1,200 mg in both China and the US, there was a corresponding increase in the ICER. Regarding cost-effectiveness, benmelstobart was advantageous when priced below $213.72 per 600 mg, considering China’s WTP threshold of $38,042.49. Similarly, in the US, the treatment was cost-effective when priced under $927.79 per 600 mg, aligning with a WTP threshold of $150,000.00.

### Sensitivity analysis

**Fig 4:**
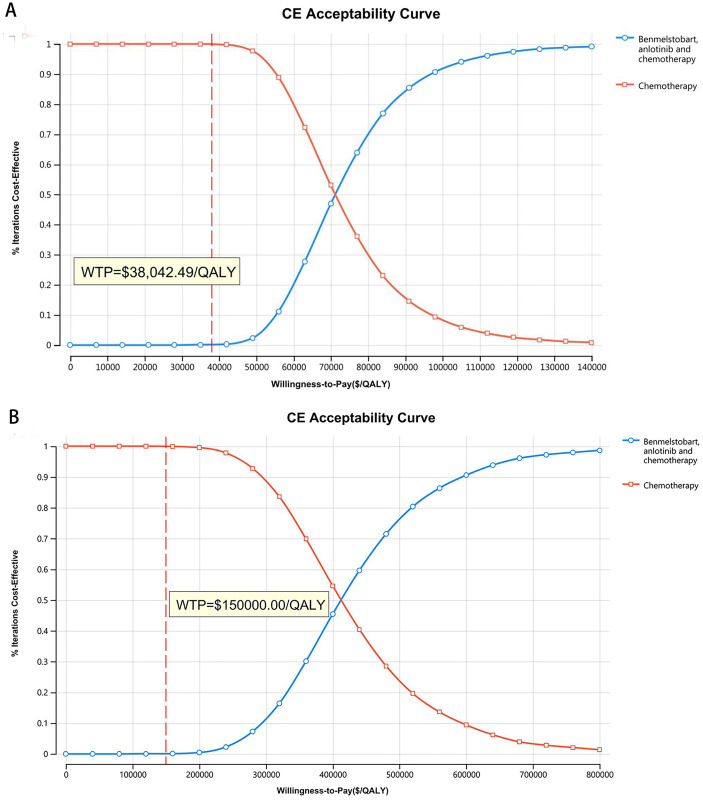
The tornado diagram of one-way sensitivity analysis. (A) China, (B) The US.

**Fig 4** presents a tornado diagram from the OWSA examining the entire population. This diagram highlights the key factors impacting base-case results: the utility value of PFS and the costs of benmelstobart and anlotinib. **Fig 4** shows that the ICER is predominantly affected by the utility value of PFS, the cost of benmelstobart, and the proportion of receiving subsequent treatment. In particular, significant discrepancies in health outcomes between the two treatment strategies ensure that variations in parameter values do not alter study outcomes.

**Fig 5:**
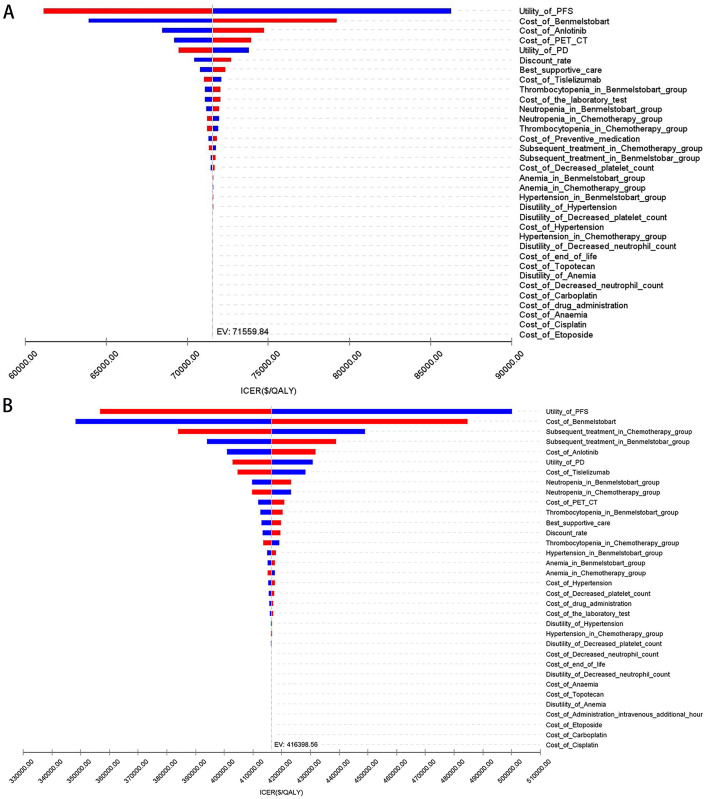
The cost-effectiveness acceptability curve. (A) China, (B) The US.

**Fig 6:**
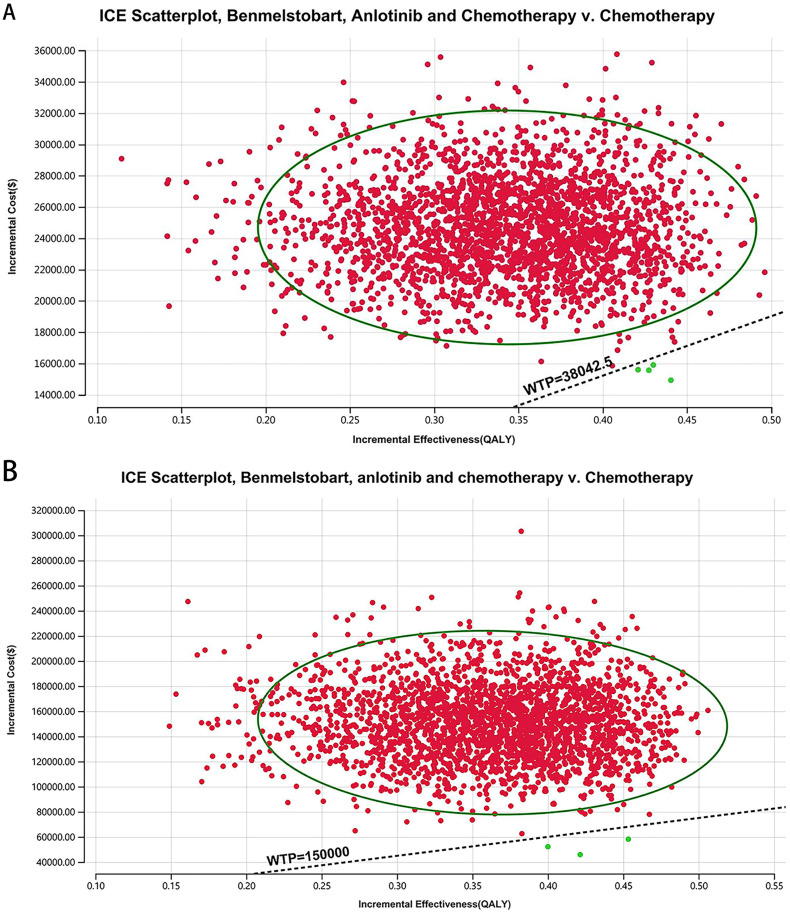
The cost-effectiveness probabilistic scatter plot. (A) China, (B) The US.

**Figs 5 and 6** show acceptability curves and probabilistic scatter plots, providing visual and practical insight into the cost-effectiveness landscape. These analytical tools are essential for decision-making, revealing the likelihood that benmelstobart and anlotinib plus chemotherapy are considered cost-effective. PSA outcomes show that the probability of cost-effectiveness is markedly low at 0.03% in China, against a WTP threshold equivalent to three times the GDP per capita ($38,042.49), and considerably higher at 0.05% in the US, based on a WTP of $150,000.00.

## Discussion

The ETER701 trial was a multicenter, double-blind, randomized, placebo-controlled, phase 3 study that compared three-drug regimens. Compared with EC alone, median PFS (6.9 months versus 4.2 months; hazard ratio 0.32) and OS (19.3 versus 11.9 months; hazard ratio 0.61) were prolonged with benmelstobart and anlotinib plus EC in patients with ES-SCLC. Although anlotinib plus EC boosted PFS and response rates compared to EC alone, the improvement in OS was not statistically significant. This could be attributed to the potential synergistic effects of anti-angiogenesis and ICI therapies in altering the tumor microenvironment. Without anlotinib, these long-term benefits might not be evident. Integrating anti-angiogenesis therapy with immunochemotherapy could be a potent and safe strategy for managing ES-SCLC.

However, our analysis indicated that combining benmelstobart and anlotinib with EC was not cost-effective as a first-line treatment within both the Chinese and US healthcare systems. Despite this being the phase III trial with the longest observed OS data for ES-SCLC, significant AEs, and substantial drug costs hinder its cost-effectiveness.

Numerous economic studies have acknowledged that while ICIs significantly improve QALYs for patients with ES-SCLC, they also introduce a significant financial burden [28,29,37–41]. These evaluations often conclude that ICIs may not be cost-effective compared to conventional chemotherapy. A recurring theme in these studies is the substantial impact of the cost of PD-L1 antibodies on health outcomes in both the US and China.

Our research adds to the growing body of evidence advocating for the use of domestically produced anticancer drugs. It has significant implications for the Chinese government, as it seeks to balance limited medical insurance funds with escalating demands for cancer treatment. In clinical decision-making for ES-SCLC, clinicians should assess the patient’s disease status along with their financial capacity and willingness to pay, prioritizing drug selection based on these factors.

Assuming that the prices for benmelstobart and anlotinib are consistent with those in China, their costs could be significantly higher in the US. However, our price simulations suggest that benmelstobart remains a favorable option when priced below $1,088.66 per 600 mg, considering the US WTP threshold of $150,000.00. This information could assist US policymakers in setting price points that would support the introduction of the benmelstobart market, while ensuring affordability. Thus, lowering the prices of ICIs could benefit a broader patient base in both China and the US. Similar economic evaluations have indicated that other therapies, such as serplulimab and adebrelimab, in combination with chemotherapy, sometimes surpass chemotherapy alone in cost-effectiveness under certain conditions [30,40].

Our sensitivity analysis revealed that the utility values for PFS and PD considerably influenced outcomes. Given the lack of quality of life (EQ-5D) and the cost per QALY data in the ETER701 trial, we relied on the literature-derived utility values for PFS and PD. Although this approach is necessary, it inherently introduces uncertainties into the modeled results. Our study distinguishes itself by using data specifically from the SCLC population rather than by generalizing findings from NSCLC studies, thereby improving the relevance and precision of our results.

This study represents the first evaluation of the cost-effectiveness of ICIs and anti-angiogenesis plus chemotherapy as first-line therapy for ES-SCLC from both the US and Chinese perspectives. However, this study has several limitations. First, the simulation model is primarily based on data from clinical trials, which introduces inherent uncertainties, a common issue with models of this nature. The alignment of the model with the survival data was confirmed by testing eight different distributions, as supported by the sensitivity analysis. Second, variability in second-line treatment options, including chemotherapy, targeted therapy, immunotherapy, and radiation, adds complexity. The absence of specific drug data in related clinical trials has led to assumptions regarding subsequent chemotherapy and best supportive care. Third, owing to the absence of price data for benmelstobart and anlotinib, the estimated US price comes from China, which might underestimate the ICER. Despite these challenges, performing a cost-effectiveness analysis using the ETER701 trial data remains feasible and offers valuable information for clinical decision-making.

## Conclusion

This analysis demonstrates that the combination of ICIs and anti-angiogenesis agents with chemotherapy does not constitute a cost-effective first-line treatment option for patients with ES-SCLC within the healthcare framework of both China and the US. This conclusion provides critical insights for healthcare decision-makers and professionals, providing substantial evidence to inform the broader integration of benmelstobart into clinical practices worldwide.

## Supporting information

S1 Table CHEERS 2022 checklist. (DOCX)

S2 Table Transition probability of Benmelstobart. (XLS)

S3 Table Transition probability of Placebo. (XLS)
